# Protective Role of C-Phycocyanin Against Secondary Changes During Sodium Selenite Mediated Cataractogenesis

**DOI:** 10.1007/s13659-014-0008-4

**Published:** 2014-04-02

**Authors:** Rasiah Pratheepa Kumari, Kumarasamy Anbarasu

**Affiliations:** Department of Marine Biotechnology, Bharathidasan University, Tiruchirappalli, 620024 Tamil Nadu India

**Keywords:** Cyanobacteria, C-phycocyanin, Cataract, Sodium selenite, Cataractogenesis

## Abstract

Age related cataract is the leading cause of blindness associated with accumulation of oxidative stress in the eye lens. The present investigation reveals the rational of the beneficial effects of the natural compound C-phycocyanin (C-PC) is beneficial when administered to rat pups to protect against the secondary effects of sodium selenite induced cataractogenesis. A single subcutaneous dose of sodium selenite (19 μmol/kg body weight) on the 10th day of postpartum is adequate to induce cataract in rat pups. Serum biochemical parameters, such as the level of electrolytes, mean activities of anti-oxidant enzymes i.e. superoxide dismutase, catalase and reduced glutathione were observed to be significantly altered during selenite induced cataractogenic process. Histopathological examination revealed signs of degradation of normal cell architecture in the liver, kidney and eye lens. Interestingly, the deleterious effects of sodium selenite toxicity were restored with the simultaneous treatment with C-PC. The results suggest that an administration of 200 mg/kg body weight of C-PC has the ability to prevent/alter the secondary changes reflected in the serum biochemical and histological modifications in rats exposed to sodium selenite. These results complement the beneficial role of C-PC of cyanobacterial origin as a efficacious anti-cataractogenic agent against sodium selenite toxicity.

## Introduction

Age related cataract is a common non-infectious disease of the eye lens, characterized by abnormalities in membrane permeation, protein aggregation, loss of glutathione redox system, imbalance in electrolytes transport, distorted calcium homeostasis, decreased enzyme activity and ATP content [1]. It accounts for more than 42 % of visual impairment and vision loss globally [2]. At the crux of the disease is the loss of endogenous reduced glutathione; however the lens epithelial cells tend to be resilient to vision loss. Further, the cataractogenic stimulus is over-ridden by a magnitude of oxidative stress which leads to the complete opacification of the lens [3]. Aging [4], smoking [5], exposure to radiation [6], and accumulation of metabolites [7] are some of the major environmental stress that cause cataractogenesis. Until recently, the surgical removal of the lens and implantation of an artificial intraocular lens is the main treatment of cataract [8]. However, surgical management inevitably result in a number of complications, such as keratopathy, postoperative glaucoma, retinal detachment, loss of accommodative power, and endophthalmitis [9].

A single dosage of sodium selenite (19 μmol/kg body weight) has been shown to be an ideal animal model to study in vivo cataractogenesis for the past decade as it reflects human senile cataract in all aspects. Sodium selenite has the potential to induce severe bilateral cataract in 10-day-old rat pups, on receiving a single subcutaneous injection of 19 μmol/kg body weight [1]. Mechanistically, exogenous selenite reacts with glutathione, the most abundant physiological sulfhydryl compound to form seleno-trisulfide which accumulates in elevated oxidative stress [10]. Selenium is an intrinsic component of glutathione peroxidase that reduces H_2_O_2_ to water at the conversion of GSH to GSSH (oxidized glutathione). However oxidative stress imparted by sodium selenite creates reactive oxygen species that induces a progressive decline of vision. Exposure of rats to sodium selenite has shown drastic changes in the hematological and biochemical parameters [11]. Sodium selenite has been documented to trigger early lenticular epithelial cell damage including loss of calcium homeostasis [12], crystallin protein breakdown [13], loss of anti-oxidant enzyme activity, accelerated apoptosis and DNA damage.

The only treatment available for cataract to date is the surgical removal of the opacified lens and its replacement with a synthetic intra ocular lens that demands a skilled surgeon and post operational issues as mentioned previously. However, recent research has been focused on the search for a prophylactic or therapeutic natural compound to treat/prevent cataractogenesis [1]. Natural compounds with beneficial properties and minimal side effects are gaining attention, in particular one such anti-oxidant; C-PC, is a soluble billiprotein present in *Spirulina platensis* and other cyanobacteria. Moreover, *S. platensis* has gained worldwide attention due to its nutraceutical attributes such as high quality protein, γ-linoleic acid, carotenoids, vitamin B_12_ and B_2_ [14]. C-PC is an accessory pigment of the light harvesting complex, which captures light energy and transfers it to chlorophyll a [15]. C-PC has been reported to possess anti-oxidant [16], neuroprotective [17], anti-inflammatory [18], hepatoprotective [19], anti-arthritic properties [20]. In our previous study, we revealed that C-PC isolated from *S. platensis* is capable of preventing the incidence of selenite induced cataractogenesis by in vitro morphological analysis and anti-oxidant enzyme modulation [21]. In this study we aim to determine the potentiality of C-PC to protect against the secondary in vivo changes imposed during selenite cataracteous conditions and to define the protective efficacy of C-PC on organs histology and serum parameters related to senile cataractogenesis.

## Results and Discussion

### Plot Profile Analysis

**Fig. 1:**
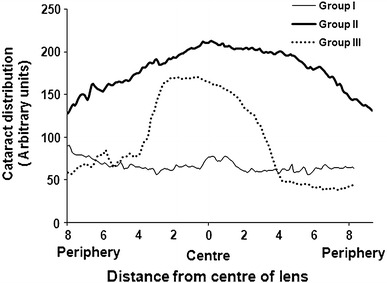
Plot profiles generated by ImageJ showed cataract distribution (*y*-axis) relative to the distance from the center of the lens (*x*-axis)

**Fig. 2:**
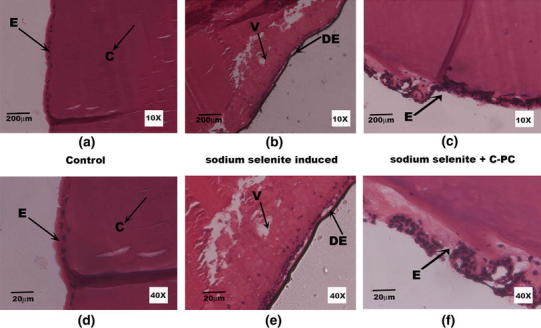
Histo-architecture of eye lens tissue: viewed under 10× magnification (**a**–**c**) and 40× magnification (**d**–**f**). Eye lens section of control group shows a organized lining of lens epithelial cells (*E*) and uniform staining of the fiber cells and cortex (*C*). Sodium selenite induced group II rat lens shows distinct vacuoles (*V*) and disorganized epithelial cells (*DE*) which is the hallmark of lens protein aggregation and opacification. However, the group III (treated with C-PC) revealed significant restoration of lenticular micro-architecture

Morphological and ImageJ software analysis (http://rsbweb.nih.gov/ij/) testified that when compared to the control animals (group I), sodium selenite administrated rats developed dense opacification of the lens (group II). Interestingly, group III revealed minimal area of cataract distribution on treatment with 200 mg/kg body weight of C-PC compared to group II rat eye lens. The plot profile analysis also indicated a minimal opacification in the rat lens treated with C-PC (Fig. 1) when compared to the Group II. The data presented suggests secondary impact on histological sections of liver and kidney caused by subcutaneous injection of sodium selenite (Fig. 1). In the epithelial cells (E), they were intact in the normal lens of control group showing distinct demarcation between the cortex (C) and nucleus (Fig. 2). Whereas prominent histological architectural alterations such as fragmented fiber cells and the cortex was observed in the cataracteous lens in group II (sodium selenite induced animals). Deep cortical vacuolization (V) was observed in group II rat lens, which is the phenomenal effect of aggregated crystallin and other lenticular proteins. With C-PC treatment (group III), the micro- architecture of the lens was significantly protected from oxidative damage induced by sodium selenite toxicity. Our results corroborate with the previous findings of Anderson et al. [22], showing aggregated proteins and vacuoles formation in the cortex region of the eye lens histological sections. The histological lesion in the cortex is possibly due to oxidative stress induced proteolysis and enhanced lipid peroxidation.

### Serum Enzyme Activities

Recently, Tripathi et al. [23] demonstrated that liver function abnormality is caused by elevated serum ALT, AST, ALP and bilirubin. Moreover, the liver is the major organ site of sodium selenite toxicity, where altered enzyme synthesis, release and catabolism of serum enzymes are major effectors and markers of cellular damage caused by oxidative stress. The serum enzymes were monitored to ascertain normal hepatic function. The mean activities of liver enzymes such as alkaline phosphatase (ALP), alanine transaminase (ALT), aspartate transaminase (AST) and gamma-glutamyl transpeptidase (GGT) were significantly elevated in sodium selenite induced group of animals (Table 1). Nevertheless, the enzyme activities were restored as to that of normal levels on simultaneous C-PC treatment (group III). A similar trend was observed with the activities of amylase, lipase and creatinine kinase (CK) (Table 1). Particularly the levels of CK, a marker enzyme of heavy metal toxicity was increased three-fold in the sodium selenite induced group when compared to the control and C-PC treated groups.

**Table 1:** The status of serum enzymes, protein content, electrolytes in control, sodium selenite induced and simultaneous C-PC treated group of animals

Parameters	Group I (control)	Group II (sodium selenite induced, cataract untreated)	Group III (sodium selenite induced, C-PC treated)
Serum enzyme activities			
ALT (U/L)	36.82 ± 1.57	432.67 ± 2.51^a,c^	39.06 ± 1.36^b,d^
AST (U/L)	19.33 ± 1.52	232.67 ± 2.67^a,c^	22.47 ± 2.08^b,d^
GGT (mg/dL)	30.33 ± 1.52	63.58 ± 0.58^a,c^	33.25 ± 2.08^b,d^
ALP (mg/dL)	38.67 ± 1.15	146.58 ± 1.00^a,c^	44.68 ± 1.52^b,c,d^
Amylase (mg/dL)	48.67 ± 1.20	82.54 ± 0.57^a,c^	57.86 ± 3.05^b,c,d^
Lipase (mg/dL)	32.67 ± 2.08	56.33 ± 3.78^a,c^	41.00 ± 1.57^b,c,d^
CK (mg/dL)	30.33 ± 1.52	146.35 ± 0.88^a,c^	45.22 ± 0.57^b,c,d^
Serum protein			
Total protein (g/dL)	7.20 ± 1.02	6.53 ± 3.80^a,c^	7.12 ± 0.17^b,d^
Albumin (g/dL)	3.72 ± 0.18	3.21 ± 1.53^a,c^	3.46 ± 0.34
Globulin (g/dL)	3.4 ± 1.57	3.02 ± 5.50^a^	3.2 ± 1.35^b^
Level of electrolytes in the serum			
Sodium (mmol/L)	140 ± 3.10	152 ± 2.13^a^	142 ± 0.36^b^
Chloride (mmol/L)	104.33 ± 0.58	100.52 ± 1.97^a,c^	103.25 ± 1.52^b^
Bicarbonate (mmol/L)	24.48 ± 0.68	36.33 ± 1.52^a,c^	26.67 ± 0.88^b,d^
Magnesium (mmol/L)	1.93 ± 0.15	3.26 ± 0.12^a,c^	2.57 ± 0.59^b,c,d^
Calcium (mmol/L)	8.1 ± 0.85	10.27 ± 0.06^a,c^	8.6 ± 0.91^b,c,d^
Phosphorous (mmol/L)	5.07 ± 0.15	2.6 ± 1.35^a,c^	3.67 ± 0.12^b,c,d^

Group I: control (received saline intraperitoneally), group II: received sodium selenite (19 μmol/kg body weight) alone, group III: received sodium selenite and treated with C-PC 200 mg/kg body weight from day 9 to 15

All values are expressed as mean ± SEM of three independent observations

^a^Statistically significant difference (*p* < 0.05) when compared with group I and others

^b^Statistically significant difference (*p* < 0.05) when compared with group II and III

^c^Statistically significant difference (*p* < 0.01) when compared with group I and others

^d^Statistically significant difference (*p* < 0.01) when compared with group II and I

**Fig. 3:**
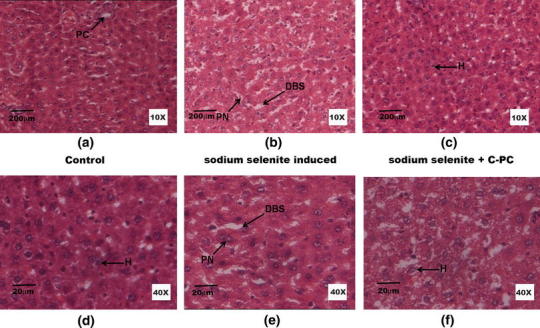
Microscopic anatomy of liver tissue: viewed under 10× magnification (**a**–**c**) and 40× magnification (**d**–**f**). Liver sections of control and treated groups showed regular morphology of the hepatocytes (*H*) including the Portal Canal (*PC*). Sections of rat liver induced with sodium selenite evidenced significant damage to the hepatocytes with the appearance of pyknotic nucleus (*PN*) and dilations in blood sinusoids (*DBS*). On treatment with C-PC the deformation were minimal, although minimal damaged hepatocytes were also observed

Histology of liver tissue obtained from control, selenite induced and C-PC treated rat pups were imaged under 10× (a–c) and 40× (d–f) magnifications. The liver section of control animals shows normal lobular architecture of the hepatocytes arranged around the portal canal (PC) with fine arrangement of Kupffer cells demonstrating normal pattern. However, in sodium selenite induced cataract group (group II) animals developed apoptotic changes such as nuclear shrinkage, pyknotic nucleus (PN) and dilations in blood sinusoid (DBS) in heptocytes. Higher magnification of liver sections from selenite induced and C-PC treated group (group III) reveals only a few degenerated hepatocytes and the conventional pattern of tissue was preserved (Fig. 3).

Release of metabolizing enzymes into the blood stream and damaged microscopic anatomy of the liver are signatures of compromised liver function exerted by sodium selenite toxicity. Amylase and lipase are pancreatic enzymes involved in the digestion and absorption of nutrients. ALP is an early indicator of hepato-cellular damage, since it can be easily mobilized into the blood at the onset of damage [24]. This anomaly is a consequence of hepatic dysfunction, however on C-PC treatment in group III, the serum components were conserved significantly. In the present study, the liver biomarker enzymes were elevated in sodium selenite induced group. This is an aftermath effect of oxidative stress exerted by sodium selenite toxicity, which certainly reflects on the liver cell morphology.

CK is one of the target enzymes for reactive oxygen species, plays a key role in energy metabolism of tissues [25] and this enzyme is moderately elevated in the serum due to liver damage. A three-fold increase in serum CK in group II animals denotes muscular and liver damage caused by oxidative stress. Nonetheless, treatment with C-PC (200 mg/kg body weight) has proven to restore the normal architecture of the hepatocytes and the levels of enzymes were maintained significantly equivalent to controls in group III. In agreement with the current findings, C-PC is reported to depress/reduce liver injury caused by carbon tetrachloride in animals [19]. This property is underpinned by the reduced rate of biotransformation of toxic species or by scavenging the reactive metabolites produced by oxidative stress [26].

### Serum Protein Metabolism

Total protein, albumin and globulin levels were assessed to probe the extent of alteration in protein metabolism. The protein content was decreased in the selenite induced group (group II) however, the levels were maintained at near normal level on simultaneous treatment of C-PC (group III) (Table 1). The serum total protein, globulin and albumin were decreased in rat pups due to the impact of sodium selenite administration due to altered intracellular protein synthesis and metabolism [25].

### Selected Serum Electrolytes

Electrolytes influence many vital body functions; a dynamic balance of electrolytes in sera imparts a major role in normal physiology and cellular territorial integrity. However, the levels of sodium, phosphorous, magnesium, bicarbonate and calcium were altered in the group II rats induced with sodium selenite (Table 1). Other electrolytes such as phosphorous and chloride were found to be imbalanced in group II rats. In the C-PC treated group (group III) such fluctuation was not observed in the levels of electrolytes, which was almost equivalent to the control group (group I). Certainly, fluctuations in electrolytes balance are pathological, whereas a compromised kidney performance is also reflected in an imbalance of electrolytes. Ca^2+^ is a vital secondary messenger and electrolyte that plays a key role in regulating Na^+^/K^+^ ATPase activity, which is a crucial transmembrane regulator of Na^+^ and K^+^ retention in the kidney and eye lens [27]. Overload of sodium, phosphorous, calcium and magnesium contributes to ATPase dysfunction and plasma electrolytes imbalance. Ca^2+^ levels in the lens is maintained at sub-micromolar range by membrane bound Ca^2+^ ATPases [28]. Researchers have reported that increased intracellular calcium activates the calcium dependent cysteine protease and calpain, leading to proteolysis of lens protein [29]. Accumulation of endogenous calcium and other electrolytes in the lenticular milieu remains the key factor for protein aggregation and opacification [30].

### General Serum Parameters

**Fig. 4:**
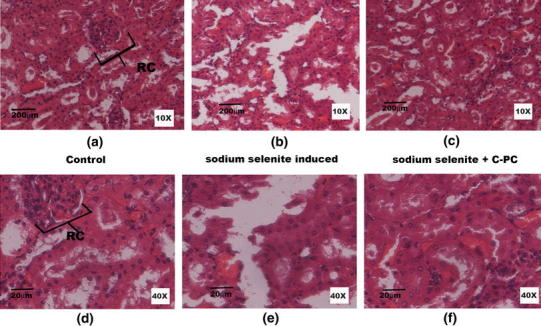
Histology of the kidney tissue: viewed under 10× magnification (**a**–**c**) and 40× magnification (**d**–**f**).The fixed sections of kidney from the control rats showed normal histology with regular Renal Corpuscles (*RC*), the functional unit of the kidneys. The kidney sections of sodium selenite induced group of animals’ revealed extensive degeneration (**b**, **e**). Group III kidney sections evidenced marginal damage to the cellular morphology (**c**, **f**) however; the normal histo-architecture was not completely restored

Serum biochemical parameters such as bilirubin, creatinine, urea, BUN, uric acid and plasma ammonia were also analyzed to evaluate the degree of renal damage induced by sodium selenite injection. All parameters mention were elevated significantly in group II except plasma ammonia (Table 2), thus conferring renal damage. Despite of the toxicity exerted by sodium selenite, concurrent treatment with C-PC controlled the levels of all the biochemical parameters to near normal levels. Due to the hemolytic property of sodium selenite [31], a significantly (*p* < 0.05) elevated bilirubin level (twofold increase) was noted in selenite-induced group of pups. Microscopic anatomy of kidney from control animals revealed regular structures of renal corpuscles (RC) (Fig. 4). The kidney sections of group II animals showed a high degree of scarification, disorganized cellular morphology, loss of brush border, vacuolization and desquamation of epithelial cells, and such lesions were not predominately encountered in the group III of animals treated with 200 mg/kg body weight of C-PC. These findings suggest that the renal damage was comparatively reduced on treating the rat pups with C-PC. Impaired kidney function has been confirmed by the disturbed histo-architecture of the tissue sections. The kidney is the primary organ of sodium selenite toxicity and its function is completely altered mainly due to it being the major route of excretion of selenite [32]. Serum concentrations of creatinine, urea, ammonia and BUN are important markers of protein catabolism and renal damage. Ostadalova [32] has recently reported that selenium toxicity has adverse effects on the ontogenic development and its retention in suckling rat pups was observed in blood, liver and kidney even after 7 days of administration. During sodium selenite induced toxicity, dehydration is inevitable, which may be the deciding factor causing the rise in serum BUN. Elevated serum levels of urea, uric acid and creatinine in sodium selenite induced pups demonstrates renal impediment [33].

**Table 2:** Status of creatinine, bilirubin, urea, BUN, plasma ammonia, uric acid in the serum and antioxidant enzyme activities in hemolysate of control, sodium selenite induced and simultaneous C-PC treated group of animals

Parameters	Group I (control)	Group II (sodium selenite induced, cataract untreated)	Group III (sodium selenite induced, C-PC treated)
Creatinine (mg/dL)	0.8 ± 0.15	0.92 ± 0.1.12^a^	0.766 ± 0.06^b^
Billirubin (mg/dL)	0.5 ± 0.25	1.1 ± 0.85^a,c^	0.63 ± 0.15^b,d^
Urea (mg/dL)	30.0 ± 0.58	42 ± 0.51^a,c^	31.67 ± 2.96^b,d^
BUN (mg/dL)	13.33 ± 1.15	19.31 ± 0.58^a,c^	13.57 ± 0.18^b,d^
Plasma ammonia (mg/dL)	40.95 ± 0.15	36.41 ± 0.57^a,c^	42.0 ± 2.64^b^
Uric acid (mg/dL)	4.13 ± 0.08	5.82 ± 0.07^a,c^	3.53 ± 0.03^b,d^
Antioxidant enzyme activities in hemolysate			
SOD (unit/g Hb)	165.94 ± 2.67	129.0 ± 0.89^a^	157.86 ± 2.37^b^
CAT (μmol of H_2_O_2_ consumed/min/g Hb)	208.56 ± 0.92	120.86 ± 1.24^a^	166.61 ± 0.77^b^
GSH (mmol/g of Hb)	237.71 ± 2.13	162.41 ± 1.73^a^	198.55 ± 1.69^b^

Group I: control (received saline intraperitoneally), group II: received sodium selenite (19 μmol/kg body weight) alone, group III: received sodium selenite and treated with C-PC 200 mg/kg body weight from day 9 to 15

All values are expressed as mean ± SEM of three independent observations

^a^Statistically significant difference (*p* < 0.05) when compared with group I and others

^b^Statistically significant difference (*p* < 0.05) when compared with group II and III

^c^Statistically significant difference (*p* < 0.01) when compared with group I and others

^d^Statistically significant difference (*p* < 0.01) when compared with group II and I

### Antioxidant Enzyme Activities

The mean SOD and CAT enzyme activities were significantly reduced in group II (selenite- induced) in comparison with group I. However, the activity was substantially retained on C-PC treatment at the significant level (*p* < 0.05). The levels of GSH was reduced considerably in the sodium selenite induced group (group II), but in the C-PC treatment in group III, the treatment restored the levels of GSH significantly to near placebo levels (Table 2). Epidemiological studies reveal that the endogenous antioxidants such as SOD, CAT, and GSH of in lens were decreased [34]. This imparts a direct correlation between the blood anti-oxidant levels and cataract incidence [35] that they could serve as systemic markers for the identification of predisposed subjects. GSH is the chief endogenous redox regulator and is drastically reduced under oxidative stress. Our results are in accordance with the earlier reports; that a reduction in anti-oxidant enzyme levels in the hemolysate of group II animals is associated evidence for enhanced oxidative stress and cataractogenesis. C-PC treatment in group III obviated the decline in anti-oxidant status in the blood (Table 2). It is probable that C-PC supplementation attenuates selenium toxicity and cataractogenesis by conservation of thiol-related processes.

## Materials and Methods

### Extraction of C-PC from *S. platensis*

*S. platensis* was cultured in Zarrouk’s media with photoperiod of 12 h light and 12 h dark at pH 9.2. C-PC was extracted by standard method as described in our previous publication [21]. All the chemicals used in the experiment were of analytical grade (HIMEDIA, Mumbai, India).

### Experimental Design

Wistar rat pups both male and female (9 day old) were used in the present study. The study protocol was approved by the institutional ethical committee (Ref No. BDU/IAEC/2012/42), Bharathidasan University, Tiruchirappalli. The pups were housed with mothers in clean polypropylene cages, maintained with a constant photoperiod of 12 h dark/light cycle. They were fed with rodent-pelleted chow and water ad libitum. These pups were randomly grouped into control and two experimental groups, comprising of eight pups per group (*n* = 8), and a total of 24 pups. On postpartum day 10, group I (control) received vehicle control saline alone, whereas group II and III were injected with sodium selenite (19 μmol/kg body weight) subcutaneously. Group III simultaneously received 200 mg/kg body weight of C-PC intraperitoneally on 9th day onwards for five consecutive days in addition to sodium selenite on 10th day subcutaneously. Subsequently after 16 days of birth, the rat pups were subjected to morphological examination of the eye for cataract development and graded according to the degree of opacity developed. On 30th day of postpartum, cataract formation was confirmed, graded and the pups were sacrificed.

The blood samples were collected in tubes (BD vacutainer), centrifuged at 5000 rpm for 20 min at 4 °C; the serum thus obtained was stored at 4 °C until further analysis. Biochemical parameters were determined using a Konelab20; a fully automated biochemical analyzer (Thermo Scientific, Finland) and the electrolytes were analyzed using a blood gas analyzer, (Siemens Rapid lab 348 Blood Gas Analyzer, Siemens Diagnostics, USA).

### Plot Profiles of Lens Images

The rat lenses were imaged with a digital camera (Power Shot A2200, Canon, India) and analyzed by ImageJ software (http://rsbweb.nih.gov/ij/) to generate plot profiles of pixel intensities and brightness along all points on a selected line. Plot profile data were exported and plotted using excel to calculate the arbitrary distribution of cataract in all the group of lenses.

### Biochemical Parameters

The following biochemical parameters were determined in the sera of control and experimental group of rats: blood urea nitrogen (BUN), urea, creatinine, uric acid, enzyme activities such as AST, ALT, ALP, GGT, amylase, lipase, CK and serum proteins (total protein, albumin and globulin). Similarly, the electrolytes such as sodium, calcium, magnesium, phosphorous, bicarbonate and chloride were determined in sera by ion selective electrodes (ISE) using blood gas analyzer.

### Preparation of Hemolysate

Anti-coagulated blood obtained from all the three groups of animals was centrifuged at 3000 rpm for 5 min at 4 °C. The packed cells were washed with isotonic buffer, followed by lysis with hypotonic buffer. The lysed cells were further centrifuged to separate the hemolysate (supernatant) [36]. The samples were stored at −20 °C until analysis.

### Antioxidant Enzyme Activities in Hemolysate

Superoxide dismutase was assayed spectrometrically in a UV–VIS spectrophotometer (Jasco V-550, USA) as described by Marklund [37] and the enzyme activity was expressed as unit/g Hb. CAT activity was assayed according to the method of Sinha [38] and the enzyme activity was expressed as micromoles of H_2_O_2_ consumed per minute per gram Hb. GSH was measured by the method of Moron [39]. The amount of GSH present in the hemolysate was expressed as millimole/gram of Hb. GSH was maintained as standard for establishing calibration curve.

### Histological Studies

Animals were sacrificed at the end of intended experimental period (30th day). The liver, kidney, eye lens tissues were separated, cleaned and diced into 0.5 cubic cm in size. Then the tissue was fixed in 10 % buffered formaldehyde (pH 7.4) at room temperature overnight. The fixed tissue was dehydrated with series of ethanol in order to remove water and infiltrated with paraffin wax. The blocks were cut into 5 μm thickness using rotary microtome and the sections were stained with Haematoxylin and Eosin. The cellular morphology and architecture was analyzed using a Nikon Microscope; Eclipse 80i.

### Statistical Analysis

All values are expressed as mean ± standard error of the mean (SEM). The statistical analysis of data was performed using one-way analysis of variance (ANOVA) followed by Student’s Newman-Keul’s test (SNK) using the Statistical Package for Social Sciences (SPSS) software package for Windows (Version 16.0; IBM Corporation, Armonk, NY, USA). The critical significance levels was considered at the level of *p* < 0.05 and *p* < 0.01.

## Conclusion

Oxidative stress is a crucial factor in the patho-physiology of sodium selenite induced toxicity and cataractogenesis [40]. The results collated in this study agree with the earlier reports that sodium selenite causes cytotoxicity, cataractogenesis and apoptosis mediated by increasing intracellular reactive oxygen species in the eye lens, kidney and liver.

Cataract and aging evoke numerous physicochemical changes in the lens proteins. We have previously reported that effects of C-PC, a billiprotein pigment in the incidence of selenite-induced cataract both in vitro and in vivo [21]. It has been documented that C-PC evades oxidative stress and related cellular damages by scavenging the free radicals and chelating metal ions [41]. The principle component tetrapyrrole chromophore present in the pigment bestows the radical scavenging property of C-PC [42]. C-PC has not only been reported for its anti-oxidant capacity but it also possess well established anti-apoptotic effects [27]. Administration of C-PC in the present study shows considerable reduction in cataractogenic process and subsequent damage to the eye lens, liver and kidney. Results also affirm the appreciable improvement of secondary serum markers of selenite toxicity and cataractogenesis. From our results it is suggested that C-PC positively modulates hepatic function by the reduction of oxidative stress and also the imbalance in electrolytes is shifted towards equilibrium. Lim [42] has established that C-PC attenuates cisplatin induced nephrotoxicity by decreasing the expression of p-ERK, p-JNK, p-p38, Bax, caspase-9, and caspase-3. Our serum biochemical and histology study further confirms that 200 mg/kg body weight C-PC reduces the impairment caused by sodium selenite toxicity probably by scavenging the free radical generated and exerting anti-apoptotic function.
